# Alternative modes of biofilm formation by plant-associated *Bacillus cereus*

**DOI:** 10.1002/mbo3.251

**Published:** 2015-03-31

**Authors:** Tantan Gao, Lucy Foulston, Yunrong Chai, Qi Wang, Richard Losick

**Affiliations:** 1 Department of Molecular and Cellular Biology, Harvard UniversityCambridge, Massachusetts, 02138; 2 Department of Plant Pathology, College of Agronomy and Biotechnology, China Agricultural UniversityBeijing, China; 3 College of Science, Northeastern UniversityBoston, Massachusetts, 02115

**Keywords:** *Bacillus cereus*, biofilm, matrix, pellicle

## Abstract

The ability to form multicellular communities known as biofilms is a widespread adaptive behavior of bacteria. Members of the *Bacillus* group of bacteria have been found to form biofilms on plant roots, where they protect against pathogens and promote growth. In the case of the model bacterium *Bacillus subtilis* the genetic pathway controlling biofilm formation and the production of an extracellular matrix is relatively well understood. However, it is unclear whether other members of this genus utilize similar mechanisms. We determined that a plant-associated strain of *Bacillus cereus* (905) can form biofilms by two seemingly independent pathways. In one mode involving the formation of floating biofilms (pellicles) *B. cereus* 905 appears to rely on orthologs of many of the genes known to be important for *B. subtilis* biofilm formation. We report that *B. cereus* 905 also forms submerged, surface-associated biofilms and in a manner that resembles biofilm formation by the pathogen *Staphylococcus aureus*. This alternative mode, which does not rely on *B. subtilis*-like genes for pellicle formation, takes place under conditions of glucose fermentation and depends on a drop in the pH of the medium.

## Introduction

Many bacteria are capable of forming multicellular communities, biofilms, which provide protection against environmental insults and facilitate symbiotic and pathogenic interactions with other organisms. The ability to form a biofilm is widespread, but the mechanisms by which community formation is achieved differs greatly between different species. In general terms, however, biofilm formation involves the coalescence of cells, often on a surface, via the production of a protective, self-produced matrix that coats the cells, sticking them together and providing a barrier against external stresses (Lemon et al. 2008). Three major components of biofilm matrix have been identified; exopolysaccharide (EPS), DNA and protein, and are used in different combinations in different bacterial species (Flemming and Wingender 2010). For example, the model Gram-positive bacterium *Bacillus subtilis* produces a biofilm that relies on an EPS component, EPS, and on a dedicated matrix protein, TasA, which is anchored on the cell wall and mediates cell–cell interactions (Lemon et al. 2008).

*Bacillus cereus* is a ubiquitous group of Gram-positive bacteria which, like *B. subtilis,* exhibit the ability to resist unfavorable conditions through biofilm formation and sporulation (Stenfors Arnesen et al. 2008). *B. cereus* is well-known as an opportunistic pathogen, particularly in connection with its frequent association with the dairy industry (Stenfors Arnesen et al. 2008). The *B. cereus* type strain ATCC 14579 has previously been investigated for its ability to form biofilms on surfaces, such as plastic, glass, and stainless steel (Oosthuizen et al. 2002; Shi et al. 2004; Hsueh et al. 2006; Vilain and Brözel 2006; Karunakaran and Biggs 2011; Lindbäck et al. 2012). However, details of the genetic basis of biofilm formation remain unclear. In fact, the only matrix component that has been described to date in *B. cereus* is extracellular DNA (Vilain et al. 2009) although there are indications that a protein component may also be present (Vilain and Brözel 2006; Vilain et al. 2009; Karunakaran and Biggs 2011).

Plant-associated strains of *B. cereus* have also been isolated. These are of particular interest since they could have bio-control applications for the prevention of plant disease (Xu et al. 2014). At least one potential bio-control strain, 0–9, has already been shown to colonize roots of wheat plants and to exhibit biofilm formation, which contributed to its bio-control efficacy (Xu et al. 2014). In this strain, a phosphotransferase PtsI was reported to be required for biofilm formation, root colonization, and effective bio-control. However, the components of the biofilm matrix produced by this strain were not investigated in detail.

Amongst Gram-positive bacteria matrix production is best understood in the model organism *B. subtilis* (López et al. 2010). Biofilm formation in *B. subtilis* begins through the receipt of specific signals by a suite of histidine kinases, KinA-E (López et al. 2009; McLoon et al. 2011; Chen et al. 2012). This results in the activation of a phosphorelay that culminates in the phosphorylation and activation of the master transcriptional regulator Spo0A. Phospho-Spo0A triggers a cascade of events, including the upregulation of an anti-repressor, SinI, which in turn antagonizes the DNA-binding activity of the dedicated biofilm repressor SinR. Derepression of SinR-controlled genes leads to the production of the two matrix components: EPS and the protein TasA. TasA is exported from the cell and processed to a mature form by the enzyme SipW before being anchored to the membrane through attachment to TapA (Branda et al. 2004; Romero et al. 2011). TasA monomers polymerize on the outside of the cell to form amyloid-like fibers which mediate cell–cell attachment (Romero et al. 2010).

We set out to investigate whether a plant-associated strain of *B. cereus,* a wheat-rhizosphere-associated strain called 905 (Wang et al. 2007), is capable of forming biofilms and, if so, how these biofilms are constructed. We wondered whether *B. cereus* 905 would form biofilms in a similar way to *B. subtilis* or if the biofilm matrix and regulatory machinery are distinct between the two species. Here, we present evidence that *B. cereus* 905 appears to exhibit two modes of biofilm formation. One mode is pellicle formation and appears to rely on orthologs of many of the genes known to be required for biofilm formation in *B. subtilis*, including two orthologs of genes for the dedicated matrix protein TasA. However, under other conditions *B. cereus* is able to form submerged, surface-associated biofilms that are not dependent on the genes required for pellicle formation. Instead, formation of these submerged biofilms is induced by low pH under conditions that result from the provision of excess glucose in the growth medium. After this work was completed Caro-Astorga et al. (2015) similarly reported a dependence of floating biofilm formation by a *B. cereus* strain on orthologs of TasA.

## Experimental Procedures

### Bioinformatic analysis

To identify homologs of known *B. subtilis* biofilm genes in *B. cereus* 905 we used the cognate gene from the *B. subtilis* 168 genome (as indicated in Table S3) to conduct nucleotide BLAST searches of the *B. cereus* 905 genome sequence (complete genome sequence unpublished; for nucleotide sequences described here see Genbank KP076259-KP076281). The percentage amino acid sequence identity of the corresponding proteins was then calculated. We used the same *B. subtilis* 168 protein sequences to identify the closest homolog amongst all known predicted proteins in the *B. cereus* group in an autonomous nonredundant protein sequence database (NCBI taxid:86661). The percentage amino acid identity between this consensus sequence and the homolog from *B. cereus* 905 was then calculated.

To identify the putative EPS biosynthetic gene cluster in *B. cereus* 905 we began by using amino acid sequences encoded in the *epsA-O* gene cluster of *B. subtilis* 168 to search for orthologs encoded by *B. cereus* 905. This led us to identify a cluster of 21 genes in the *B. cereus* genome which we then compared by protein BLAST to a predicted EPS gene cluster described in the sequenced *B. cereus* strain ATCC 14579 (Ivanova et al. 2003).

### *B. cereus* strains and plasmids

*B. cereus* strains and the plasmids used in the manipulation of these strains are described in Table S1. The *B. cereus* isolate 905 used in this study, which has been described previously (Wang et al. 2007), was isolated from wheat rhizosphere. This strain was assigned to the *B. cereus* group on the basis of its 16S rRNA gene sequence, by comparison to sequences in the NCBI database (data not shown).

### Media and growth conditions

*B. cereus* was routinely grown at 25°C or 37°C in LB broth or on LB agar (BD, Franklin Lakes, NJ, USA) supplemented, when required, with erythromycin (5 *μ*g mL^−1^), tetracycline (10 *μ*g mL^−1^) and kanamycin (50 *μ*g mL^−1^). For biofilm assays Tryptic Soy Broth (TSB) (EMD Millipore, Billerica, MA, USA), sterilized by filtration (autoclaving was found to reduce biofilm formation by *B. cereus* 905), was used. TSB broth was supplemented, where indicated, with 1% glucose, proteinase K (0.1 mg mL^−1^) (Omega Bio-Tek, Norcross, GA, USA) or DNaseI (5 U mL^−1^) (Qiagen, Valencia, CA, USA). Other media utilized were TSB without glucose (Peptone from casein [BD] 17 g L^−1^, Peptone from soymeal (Amresco, Solon, OH, USA) 3 g L^−1^, NaCl (Sigma, St. Louis, MO, USA) 5 g L^−1^ and dipotassium hydrogen phosphate (Macron, Center Valley, PA, USA) 2.5 g L^−1^), BHI (EMD Millipore), MSgg (Branda et al. 2001), LBGM (Shemesh and Chai 2013) and M9 (De Kievit et al. 2001). *Escherichia coli* DH5*α* was grown at 37°C in LB medium and on LB agar supplemented, when required, with ampicillin (100 *μ*g mL^−1^), kanamycin (50 *μ*g mL^−1^) or tetracycline (10 *μ*g mL^−1^). The pH of biofilm cultures was measured by spotting 20–30 *μ*L on pH-indicator strips (range 5.0–10.0) (EMD Millipore).

### *B. cereus* pellicle formation assay

*B. cereus* cells were grown in LB broth at 37°C to stationary phase. For pellicle formation, 2 *μ*L of the cells were mixed with 2 mL of MSgg broth in 24-well plates (353047; Corning, Tewksbury MA, USA). Plates were incubated at 30°C for 3 days. All images were taken using a Sony (New York, NY, USA) NEX-5 digital camera.

### Construction of marked deletion mutants in *B. cereus* 905

The *spo0A* mutant strain TG001 was constructed using the following procedure. A kanamycin resistance gene (Km) was amplified by polymerase chain reaction (PCR) with Platinum Pfx DNA Polymerase (Life Technologies, Grand Island, NY, USA) from plasmid pMarA (Breton et al. 2006) using primers Km-F and Km-R (All primer sequences are shown in Table S2). The resulting fragment was digested with BamHI/PstI and cloned into pEBS (Wang et al. 2007), which had also been digested with BamHI/PstI, generating pEBSK. Regions of 967-bp upstream and 1094-bp downstream of the *spo0A* gene were amplified from *B. cereus* 905 genomic DNA using the primers spo0A-Up-F/spo0A-Up-R and spo0A-Dn-F/spo0A-Dn-R, respectively. These fragments were digested with *Xba*I/BamHI and PstI/XhoI, respectively, and were cloned into the pEBSK plasmid to create pEBSKΔ*spo0A*. A 3072-bp Up-Km-Dn fragment was amplified by PCR from pEBSKΔ*spo0A* using primers spo0A-F and spo0A-R. The fragment was digested with BglII and MluI, and cloned into a temperature-sensitive *E. coli* and *Bacillus* shuttle vector pMAD (Arnaud et al. 2004) that had also been digested with BglII and MluI, generating pMADΔ*spo0A*. The construct was confirmed by PCR and Sanger sequencing. The pMADΔ*spo0A* plasmid was mobilized into *B. cereus* 905 by electroporation. Transformants were obtained after incubation for 2 days at 30°C on LB plates containing 1 *μ*g mL^−1^ erythromycin and 40 *μ*g mL^−1^ of X-Gal. Allelic replacement of the temperature-sensitive pMADΔ*spo0A* in *B. cereus* 905 was achieved through the following procedure. Single crossover integrants were induced by growth overnight at 42°C in the presence of erythromycin. Double crossover events were promoted by two 12 h rounds of growth in liquid culture at 25°C. Erythromycin sensitive clones were isolated and mutants identified by colony PCR. All mutants were subsequently confirmed by PCR amplification and Sanger sequencing to verify the presence of the *spo0A*::Km allele on the chromosome (data not shown).

Replacement of other genes in the *B. cereus* 905 chromosome with the kanamycin resistance cassette was carried out as described for Δ*spo0A,* with the exception that the primers shown in Table S2 were used to amplify the upstream and downstream homologous regions for each gene.

### Construction of an *eps* unmarked deletion mutant in 905

An in-frame, unmarked deletion of the first six genes of the *eps* operon in the *B. cereus* 905 genome was constructed essentially as described in (Arnaud et al. 2004). Briefly, a construct was generated in which ∼900 bp regions of the *B. cereus* 905 genome, flanking the deletion site, were amplified by PCR and were introduced by Gibson Assembly (Gibson et al. 2009) into pMAD, linearized with NcoI and MluI. The resulting plasmid was mobilized into *E. coli* DH5*α* and transformants were selected for growth on ampicillin. The resulting construct was confirmed by PCR and Sanger sequencing. The construct was subsequently mobilized into *B. cereus* 905 by electroporation and transformants were selected on 1 *μ*g mL^−1^ erythromycin and 40 *μ*g mL^−1^ X-Gal at 30°C. Allelic replacement and mutant confirmation were carried out as described above.

### Construction of complementation strains

The plasmid pGFP78 carries a constitutive promoter (F78) that was selected from a *B. subtilis* ISW 1214 genomic DNA library, for the property of driving high-level expression of green fluorescent protein (GFP) (Q. Wang, pers. comm.). The GFP open reading frame (ORF) was removed from this vector by digestion with *Xba*I and *Hin*dIII. The respective wild-type gene from *B. cereus* 905 was amplified by PCR using gene-specific primers (shown in Table S2) and was ligated to the *Xba*I and *Hin*dIII sites of the cut plasmid, to generate each respective complementation plasmid (as shown in Table S1). Construction of the *ΔsinI* complementation construct was altered from the above in that the complete 135-bp *sinI* gene of *B. cereus* 905 was amplified by PCR with primers sinI-F-C and sinI-R-C, and ligated to pHY78 digested with *Hin*dIII, to generate p78*sinI*. All plasmids were confirmed by PCR and Sanger sequencing. Plasmids were subsequently mobilized into the corresponding *B. cereus* mutants by electroporation and transformants selected with tetracycline.

### *B. cereus* submerged biofilm assay

Submerged biofilm formation was quantified essentially as described previously (Foulston et al. 2014). *B. cereus* 905 was grown from a single colony in 3 mL LB overnight at 25°C with aeration. For biofilm growth, cells were diluted 1 in 1000 into fresh medium (typically filter sterilized TSB supplemented with 1% glucose (TSBG) or as specified in the text) and 200 *μ*L was aliquoted into a Nunc™ MicroWell™ 96-Well Microplate (167008; Thermo Scientific, Tewksbury, MA, USA). Typically the starting OD_600_ was recorded using a BioTek Synergy II plate reader (BioTek Instruments, Winooski, VT, USA). Plates were incubated statically at 37°C for 24 h. To quantify biofilm formation, the medium in each well was carefully removed with a multichannel pipette and transferred to wells of a new 96-well microtiter plate. The biofilms were then washed once with 200 *μ*L phosphate-buffered saline pH 7.5 (PBS) using a multichannel pipette and the wash transferred into wells of a new 96-well microtiter plate. The biofilms were then resuspended in 200 *μ*L PBS using a multichannel pipette. The OD_600_ of each fraction was then recorded using a BioTek Synergy II plate reader. To remove background absorbance the starting OD_600_ was subtracted from the values for each medium sample, and the absorbance of a well containing PBS alone was subtracted from the wash and resuspended biofilm values. Replicate wells (*n* ≥ 4) were averaged and a standard deviation calculated. Each strain was tested independently at least three times.

For crystal violet assays biofilm cultures were grown in 12-well plates (353043; Falcon). After 24 h of growth at 37°C the medium in each well was gently removed with a pipette and each well was washed twice with 1 mL of PBS to remove unattached bacteria. The plates were stained with a 0.1% crystal violet solution (Sigma) for 30 min and then washed once with 1 mL of PBS. Plates were air dried before photographing.

To test biofilm formation of *B. subtilis* 3610 the strain was grown overnight in LB at 25°C before diluting 1 in 1000 into fresh medium (as indicated in figures and text) and growing in a Nunc™ MicroWell™ 96-Well Microplate (167008; Thermo Scientific) for 24 h at 37°C. Pellicles formed were photographed before being manually removed with a plastic pipette tip to visually determine whether any biomass was surface associated.

## Results

### Candidate biofilm genes in *B. cereus* 905

Genome sequences from members of the *B. cereus* group of bacteria encode homologs of many of the genes known to be important for biofilm formation in *B. subtilis*, such as the master regulator *spo0A*, the dedicated biofilm regulatory genes *sinI* and *sinR,* and two *tasA*-like genes (Pflughoeft et al. 2011; Fagerlund et al. 2014). In *B. subtilis* a single copy of *tasA* is located in an operon with *sipW* and *tapA* (*yqxM*) (Branda et al. 2004). By contrast *B. cereus* strains appear to possess two genes with predicted products with similarity to TasA (Pflughoeft et al. 2011). However, one of these two gene copies has frequently been annotated in *B. cereus* genome sequences as *calY* due to the similarity of the predicted amino acid sequences to that of camelysin, a proteolytic enzyme purified from *B. cereus* strain DSM 14729 (Grass et al. 2004; Pflughoeft et al. 2011). It has not yet been determined whether these proteins can also function like TasA, playing a structural role in the biofilm matrix. However, the amino acid similarity shared by the predicted proteins of the *calY* genes with TasA and the conserved synteny of the *B. cereus tasA* and *calY* genes with both *sipW* and *sinI/sinR,* suggests that both of these proteins are functional equivalents of *B. subtilis* TasA. Furthermore, in both *B. anthracis* and *B. thuringiensis* these genes are under the control of the biofilm repressor SinR as is *tasA* in *B. subtilis* (Pflughoeft et al. 2011; Fagerlund et al. 2014). Interestingly, despite the presence of two homologs of *tasA* we were unable to identify a homolog of *tapA* in the *B. cereus* group. This might suggest either that a TapA function is not required in *B. cereus* or, conceivably, that one of the two TasA homologs can functionally substitute for TapA.

**Figure 1:**
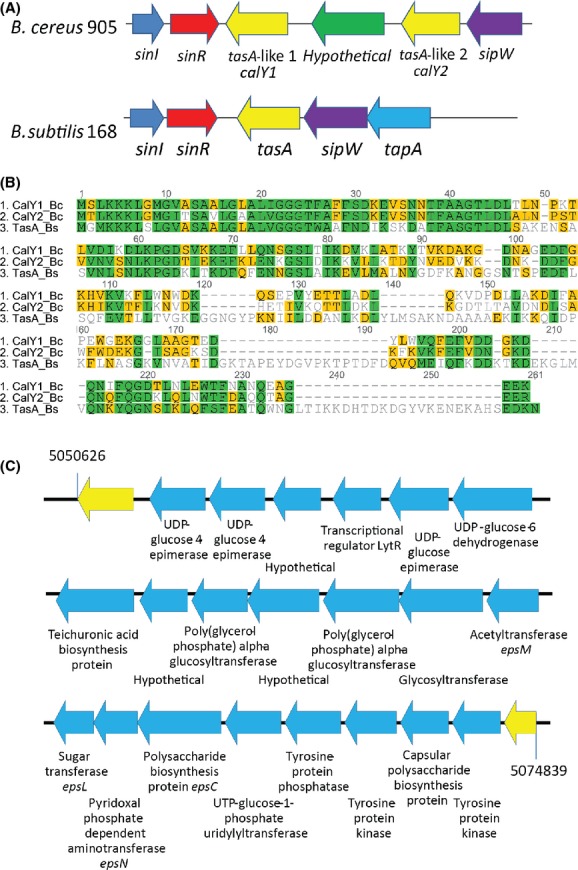
Putative biofilm genes in *Bacillus cereus* 905. (A) The arrangement of genes in the region of *sinI*-*sinR* in *B. cereus* 905 is shown with the comparable region of the *Bacillus subtilis* 168 chromosome. (B) An alignment of the amino acid sequences of; TasA from *B. subtilis* 168 (TasA_Bs), and CalY1 (CalY1_Bc) and CalY2 (CalY2_Bc) from *B. cereus* 905, produced using Geneious 7.0.4 (created by Biomatters). Green highlighting indicates 100% similarity, yellow indicates 60–80% similarity and white indicates less than 60% similarity. (C) The arrangement of genes in the putative exopolysaccharide operon of *B. cereus* 905. Genes predicted to be involved in EPS biosynthesis are highlighted in blue with flanking genes in yellow. The predicted function of the encoded protein, based on amino acid similarity, is shown below each gene.

Like other members of the *B. cereus* group, homologs of genes involved in *B. subtilis* biofilm formation were identified in the *B. cereus* 905 genome sequence (Genbank KP076259-KP076281) (Table S3). Although the identity of the predicted products of these genes between *B. subtilis* and *B. cereus* varied from 35% to 82%, the gene synteny was similar. For example, as in *B. subtilis* the *sinI-sinR* gene pair was located near the two TasA-like genes, named *calY1* and *calY2,* and *sipW* (Fig.1A). One of the *tasA*-like genes (*calY2*) occurs downstream of *sipW* while the other is nearby (Fig.1A). The overall amino acid identity between TasA from *B. subtilis* and CalY1 and CalY2 from *B. cereus* 905 was about 35% and the similarity was 51%. Several amino acid motifs were conserved between the three proteins, particularly within the N-terminal half of the proteins (Fig.1B).

Another important feature of *B. subtilis* biofilm formation is EPS production from the biosynthetic gene cluster *epsA-O* (Branda et al. 2001). We identified in the *B. cereus* 905 genome sequence, a 21-gene cluster with predicted products with many of the hallmarks of EPS biosynthetic machinery (Fig.1C and Table S4), such as glycosyltransferases and a tyrosine kinase (EpsAB) known to regulate EPS biosynthesis in *B. subtilis* (Elsholz et al. 2014). However, the overall similarity of this region to the corresponding *eps* gene cluster (15 genes) in *B. subtilis* was relatively low and only a few genes appeared to be conserved (for example; *epsC*,*epsN*, and *epsL*). We also compared the *B. cereus* 905 gene cluster to a previously predicted capsular EPS gene cluster from the strain ATCC 14579 (BC5279–BC5263) (Ivanova et al. 2003) (Table S4). Just as with *B. subtilis,* however, many of the encoded proteins from the *B. cereus* 905 cluster were not conserved in *B. cereus* ATCC 14579. Only those genes likely to be involved in regulation of EPS biosynthesis such as tyrosine kinases and a LytR-type regulator shared high amino acid identity between the two strains and many genes did not appear to have an equivalent protein encoded by the other strain (Table S4). This suggests that EPS biosynthesis is not well-conserved among *B. cereus* strains and might reflect the use of different forms of EPS by different species and strains.

### *B. cereus* pellicle biofilm formation

**Figure 2:**
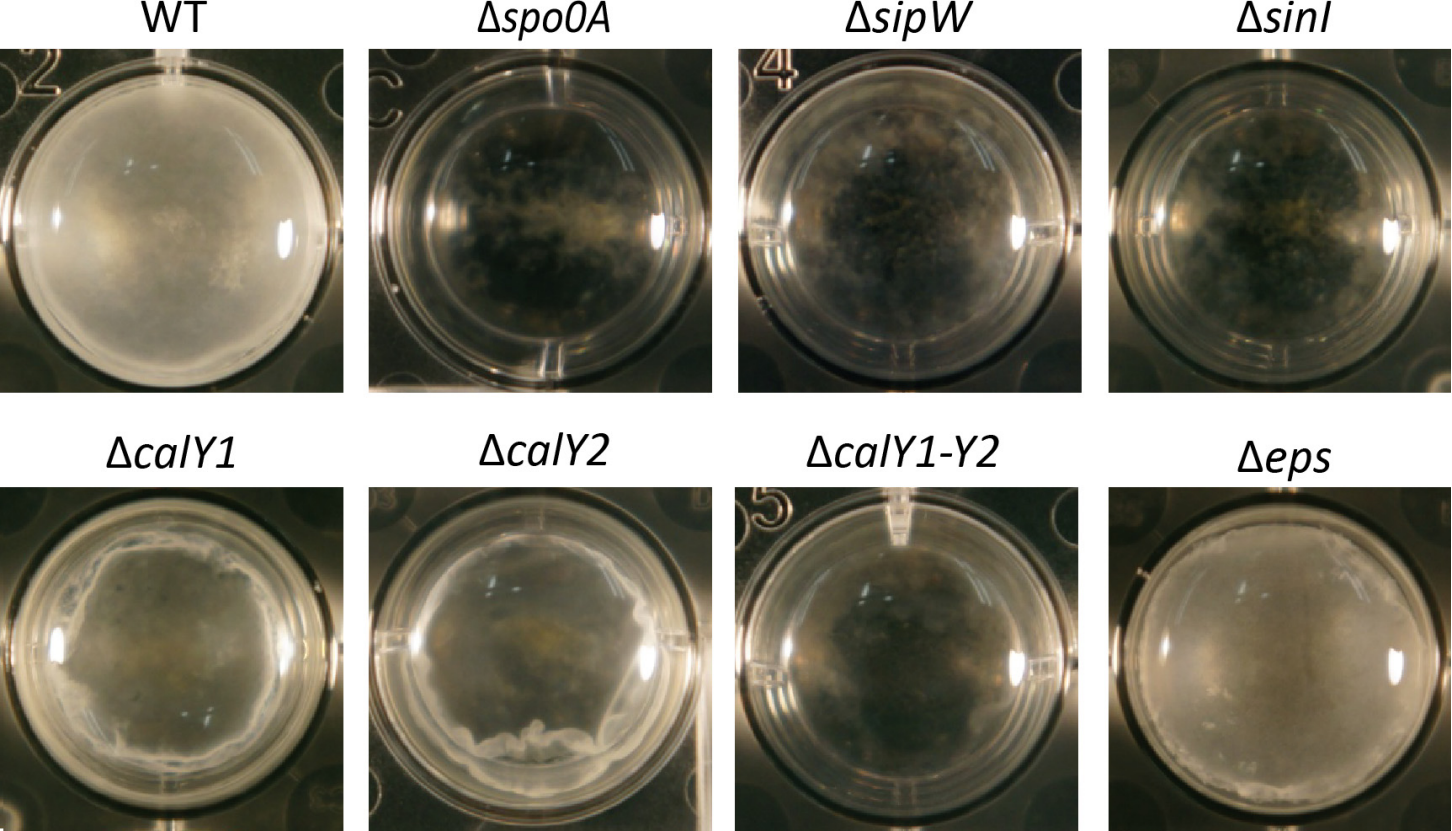
Pellicle Formation by *Bacillus cereus* 905. *B. cereus* 905 wild-type and deletion mutants, as indicated, were grown in MSgg medium for 3 days at 30°C in a 24 well plate and images were taken.

MSgg medium is commonly utilized for the formation of biofilms in *B. subtilis*. In MSgg medium, *B. subtilis* cells form floating biofilms (pellicles) at the air–liquid interface (Branda et al. 2004). *B. cereus* 905 similarly formed a pellicle-like structure when grown in MSgg (Fig.2). *B. cereus* pellicles were not as thick or wrinkled as those made by *B. subtilis* but instead were thin films coating the surface of the liquid.

Are any of the genes that contribute to biofilm formation by *B. subtilis* important for pellicle formation by *B. cereus* 905? To investigate this, mutant strains of *B. cereus* 905 were constructed in which candidate genes were deleted from the chromosome. The deletion mutant strains were then grown alongside wild-type *B. cereus* 905 in MSgg medium at 30°C and visually observed for the formation of pellicles after 3 days incubation.

In *B. subtilis*,*spo0A* encodes a master transcriptional regulator of development that is required to initiate both biofilm formation and sporulation. A *B. cereus* 905 *spo0A* deletion mutant was similarly unable to make a pellicle biofilm (Fig.2). To confirm the role of this gene as a pleiotropic regulator of development in *B. cereus* we also examined the ability of this strain to generate spores. By microscopy we were unable to visualize any phase-bright spores being formed by the mutant*,* unlike the wild-type strain in which almost all cells were able to sporulate (Fig. S1). To confirm that the phenotype of the *spo0A* mutant was not the result of a second-site mutation we introduced a copy of the wild-type *spo0A* gene into the mutant strain on a plasmid (p78*spo0A*). This strain was restored in the ability to form a pellicle (Fig. S2).

In *B. subtilis,* SinI is required to induce the derepression of biofilm matrix gene expression and is thus essential for biofilm formation. A *B. cereus sinI* deletion mutant was similarly unable to make a pellicle biofilm (Fig.2). We introduced a copy of the wild-type *sinI* gene into the mutant strain on a plasmid (p78*spo0A*). This strain was restored in the ability to form a pellicle (Fig. S2).

Two homologs of *tasA*, the product of which is the *B. subtilis* matrix protein, were identified in *B. cereus* 905. Despite the low level of amino acid sequence identity between the predicted proteins of these genes and TasA and their previous annotation as protease-encoding genes, we wondered whether CalY1 and CalY2 were required for pellicle formation. In *B. subtilis* TasA is processed by the signal peptidase SipW, which is thus also essential for biofilm formation. Deletion of *sipW* in *B. cereus* inhibited pellicle formation (Fig.2). However, deletion of *calY1* or *calY2* individually caused only a partial defect in pellicle formation, generating weaker pellicles that did not float on the liquid surface but could be seen at the bottom of the well (Fig.2). These phenotypes could be complemented in *trans* by providing a wild-type copy of the respective gene (Fig. S2). To determine whether *calY1* and *calY2* have partially redundant functions in *B. cereus* pellicle formation we were interested to determine the phenotype of a double mutant. However, due to the challenges associated with genetically manipulating *B. cereus,* we instead chose to delete the entire region spanning the two genes. It should be noted that this includes another hypothetical open reading frame that lies between *calY1* and *calY2* (Fig.1A). The resulting *calY1-Y2* deletion mutant was completely unable to form a pellicle (Fig.2). Despite the presence of an ORF of unknown function between the two genes, the most likely interpretation of this result, given the partial phenotypes of the single mutants, is that *calY1* and *calY2* display redundant functions in pellicle formation.

Finally, to assess the contribution of EPS to pellicle formation in *B. cereus* 905 we deleted the first six genes of the putative EPS gene cluster (Table S4 and Fig.1C). This mutant was, however, unaffected in its ability to form pellicles (Fig.2). This might suggest either that these six genes are unnecessary for EPS production or that the EPS product of this gene cluster is not a major component of *B. cereus* 905 biofilms.

### Conditions promoting surface-associated *B. cereus* biofilm formation

**Figure 3:**
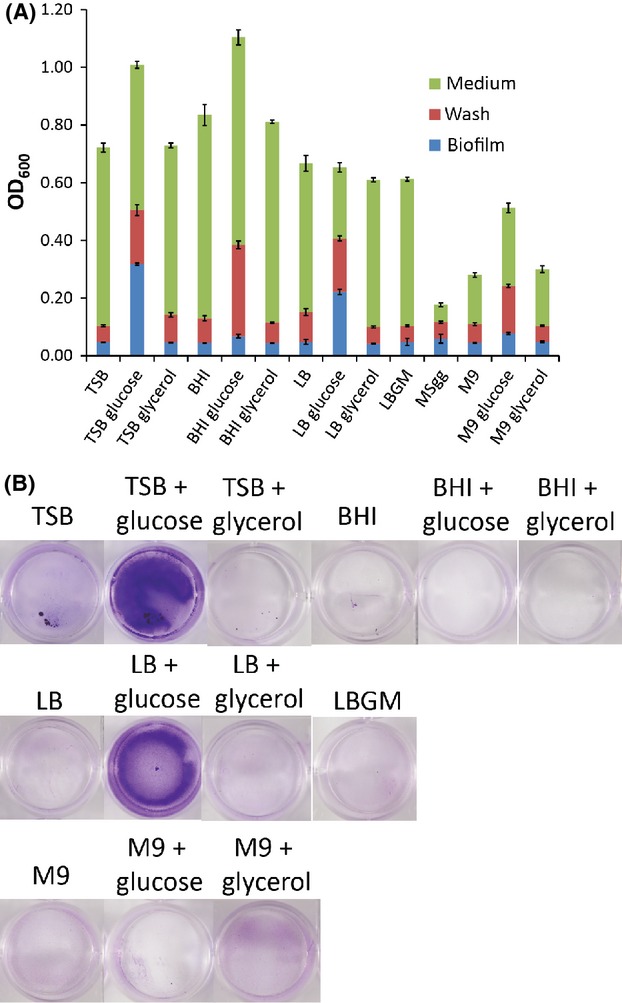
Submerged biofilm formation by *Bacillus cereus* 905 in different growth media. (A) *B. cereus* 905 was grown in the indicated growth media in wells of a 96-well tissue culture plate at 37°C for 24 h. The medium was removed and the OD_600_ measured (medium). The well surfaces were washed with PBS and the OD_600_ of the washes measured (wash). Finally cells on the surface of the well were removed by vigorous resuspension in PBS and the OD_600_ measured (biofilm). Values shown are the average of three independent experiments with error bars indicating the standard deviation. (B) *B. cereus* 905 was grown in the indicated growth media in wells of a 12-well tissue culture plate at 37°C for 24 h. The medium was removed and biofilms were washed twice with PBS. The plates were stained with a 0.1% crystal violet solution for 30 min and then washed once with 1 mL of PBS. Plates were air dried before photographing. PBS, phosphate-buffered saline.

We next wondered whether *B. cereus* can form biofilms under other growth conditions. We observed that when grown in complex media *B. cereus* formed submerged biofilms that were associated with the bottom surface of the wells of the microtiter plate in which they were grown, rather than floating as a pellicle. *B. cereus* formed measurable surface-associated biofilm in complex media such as TSB and LB but only when excess glucose was provided in the medium (Fig.3). Approximately 20–30% of total biomass (see Experimental Procedures for details of biofilm quantification) was found to associate with the surface of tissue culture plates when cells were grown in TSB with excess glucose. However, in the absence of glucose (TSB) or in the presence of glycerol as a carbon source, biofilm biomass was reduced fivefold to ∼6%. *B. cereus* grew poorly or did not make a substantial surface-associated biofilm in the defined or minimal media tested (Fig.3).

### Submerged biofilm formation requires low pH

**Figure 4:**
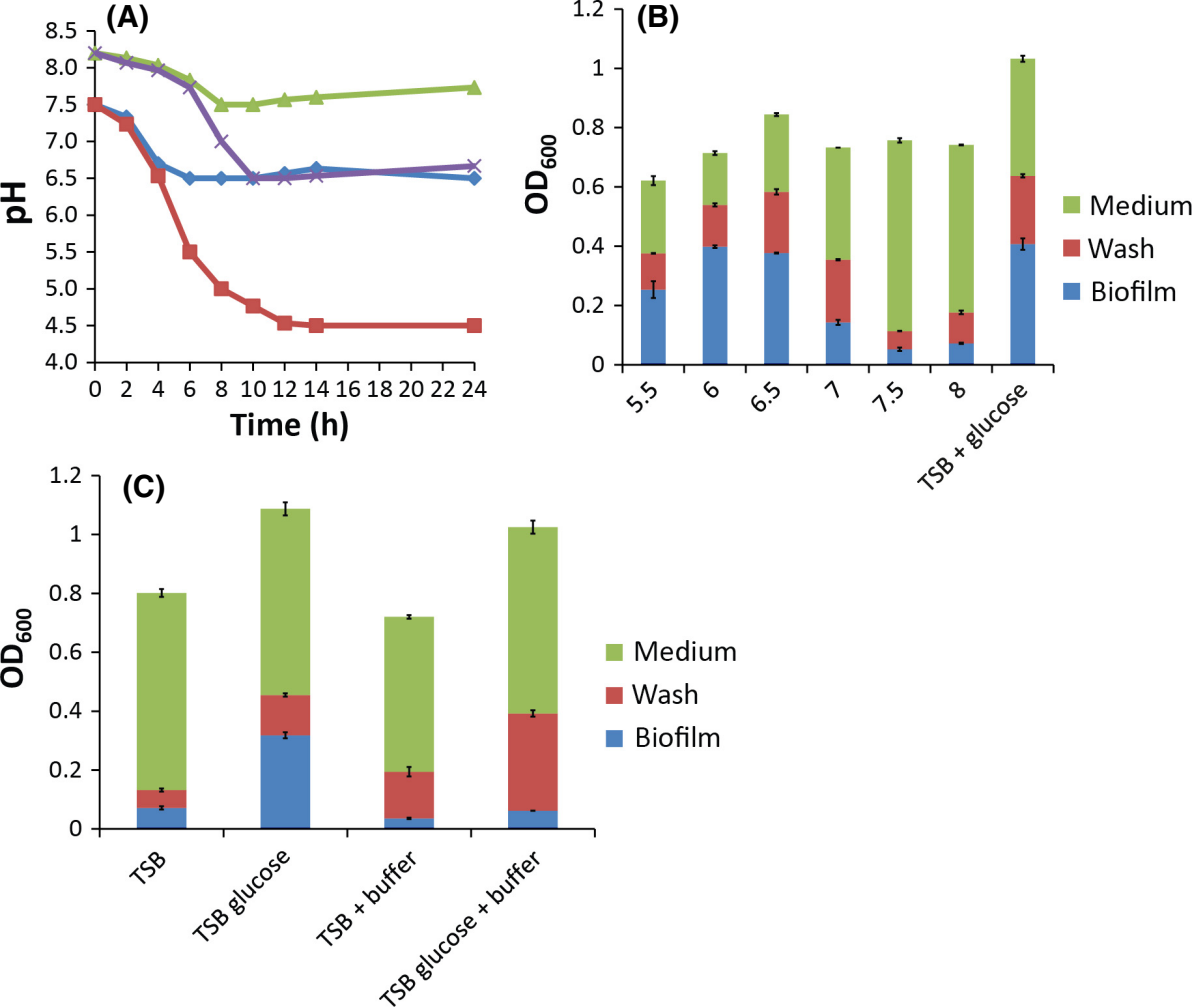
Biofilm formation by *Bacillus cereus* 905 depends on low pH. (A) *B. cereus* 905 was grown in wells of a 12-well tissue culture plate at 37°C for 24 h. At the indicated time points 30 *μ*L medium was removed and spotted on pH paper to determine the pH. Growth media were TSB (blue ◊), TSB supplemented with 1% glucose (red □), TSB supplemented with 200 mmol/L sodium phosphate buffer (green Δ), and TSB supplemented with 1% glucose and with 200 mmol/L sodium phosphate buffer (purple X). (B) *B. cereus* 905 was grown in TSB medium, to which acetic acid had been added to give the indicated starting pH values, in wells of a 96-well tissue culture plate at 37°C for 24 h. Biofilm formation was quantified as Figure3. Values shown are the average of three independent experiments with error bars indicating the standard deviation. (C) *B. cereus* 905 was grown in the indicated media with and without 200 mmol/L disodium phosphate (buffer) in wells of a 96-well tissue culture plate at 37°C for 24 h. Biofilm formation was quantified as Figure3. Values shown are the average of three independent experiments with error bars indicating the standard deviation.

The requirement of *B. cereus* for excess glucose in order to form submerged biofilms in complex medium was reminiscent of biofilm formation by another Gram-positive bacterium, *Staphylococcus aureus*. *S. aureus* ferments excess glucose to generate mixed mild acids during biofilm growth, which subsequently decrease medium pH to around 4.5–5. This pH decrease is crucial for *S. aureus* biofilm formation and preventing the pH decrease by supplying a buffer prevents biofilm formation (O'Neill et al. 2008; Foulston et al. 2014). We wondered whether a similar process occurs when *B. cereus* is grown in the presence of excess glucose. To investigate this, *B. cereus* was grown under biofilm conditions and the pH of the growth medium was measured over 24 h of biofilm formation. When *B. cereus* was grown in TSB supplemented with 1% glucose the pH of the growth medium decreased to pH 4.5 over the 24 h period (Fig.4A). By contrast, in the absence of glucose, although there was an initial small dip in medium pH, the final pH was 6.5. Since biofilm formation is associated with the provision of glucose, we conclude that a decrease in medium pH contributes to the ability of *B. cereus* to form a biofilm in TSB.

To further test our hypothesis we next used acetic acid to manually decrease the pH of TSB in the absence of glucose. When the starting pH of the growth medium was at or lower than pH 6.5 *B. cereus* displayed robust biofilm formation (Fig.4B) and when measured after 24 h the final pH of the medium under these conditions was pH 5. By contrast, little to no biofilm was seen when the starting pH was pH 7 or greater even though overall cell biomass was unaffected (Fig.4B). The pH of these cultures, when measured after 24 h, was greater than pH 6.5. Our results were recapitulated when observing biofilm formation by crystal violet staining (Fig. S3). This indicates that low pH contributes to biofilm formation and that the source of this pH drop is likely excess glucose in the medium.

We further established that a decrease in pH is required for biofilm formation by *B. cereus* in TSB by preventing the pH decrease with a buffer, 200 mmol/L disodium phosphate. This buffer was effective in preventing the decrease in pH observed when *B. cereus* was grown in TSB supplemented with glucose (Fig.4A). Sodium phosphate buffer prevented biofilm formation by *B. cereus* while not affecting overall biomass (Fig.4C). Our results were recapitulated when observing biofilm formation by crystal violet staining (Fig. S3). In toto, these results indicate that, like *S. aureus*,*B. cereus* experiences a significant decrease in medium pH during biofilm formation and that this pH decrease is crucial for the formation of a submerged biofilm.

### Determining the composition of the biofilm matrix

**Figure 5:**
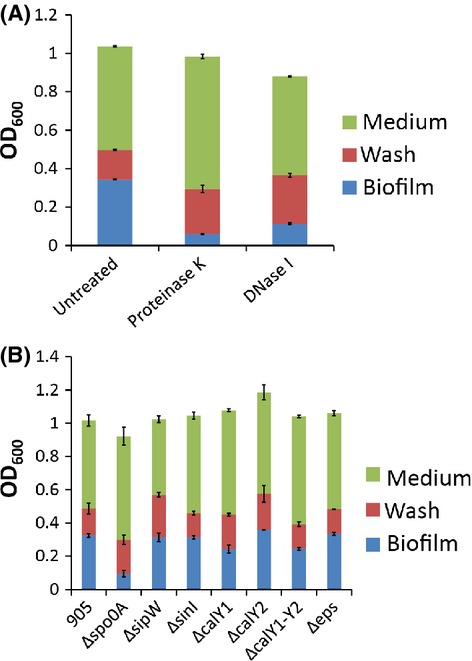
Factors contributing to *Bacillus cereus* biofilm matrix. (A) *B. cereus* 905 was grown in TSB supplemented with 1% glucose in wells of a 96-well tissue culture plate at 37°C for 24 h. At inoculation either proteinase K or DNase I was added as indicated. (B) *B. cereus* 905 wild-type and the indicated deletion strains were grown in TSB supplemented with 1% glucose in wells of a 96-well tissue culture plate at 37°C for 24 h. In both cases biofilm formation was quantified as in Figure3. Values shown are the average of three independent experiments with error bars indicating the standard deviation.

We next investigated what might be responsible for holding cells together in the submerged *B. cereus* biofilm; in other words, what was the composition of the matrix? We first investigated whether protein and/or DNA are important matrix components, as they are in *S. aureus* submerged biofilms (Foulston et al. 2014), by preventing their accumulation outside cells with protease and nuclease treatment. When either proteinase K or DNase I were added to the cultures, from the start of the incubation, biofilm formation was inhibited, without a significant effect on growth (Fig.5A). Proteinase K was more effective at preventing biofilm formation than DNase I, reducing the percentage of the population present in the biofilm fraction by 80%, whereas DNase I caused only a 60% reduction. This suggests that both protein and DNA play an important role in holding cells together in a biofilm and thus contribute to the matrix. Additionally, we tested the importance of EPS for submerged biofilm formation by quantifying biofilm formation by a mutant deleted for the first six genes of the eps operon (Δ*eps*), as described above. This mutant displayed wild-type levels of submerged biofilm formation (Fig.5B) suggesting that these genes are not essential for the formation of a submerged biofilm by *B. cereus*.

Since homologs of genes important for *B. subtilis* biofilm formation were found to be essential for pellicle biofilm formation we wondered whether these genes can also contribute to the ability of *B. cereus* to form surface-associated submerged biofilms. A *spo0A* deletion mutant formed biofilms poorly by comparison to the wild-type strain (Fig.5B), displaying only about 30% of the biomass of the wild-type biofilm. Biofilm formation was restored to wild-type levels by provision of the wild-type gene in *trans* (Fig. S4).

By contrast deletion of *sipW*,*sinI,* and *calY2* caused little to no biofilm defect in *B. cereus* 905 (Fig.5B). Deletion of *calY1*, caused a small but reproducible decrease in biofilm formation, reducing the percentage of biofilm biomass to 70% of wild-type biofilm (Fig.5B). This strain was restored to wild-type levels of biofilm formation by provision of the wild-type gene in *trans* (Fig. S4). In this case, unlike for pellicle formation, CalY1 and CalY2 do not appear to act redundantly since a deletion of both genes together (Δ*calY1-Y2*) recapitulated the phenotype of the single *calY1* mutant (Fig.5B).

### *B. subtilis* 3610 does not form submerged biofilms

Since *B. cereus* 905 forms entirely submerged and surface-associated biofilm when grown in complex media in the presence of glucose we wondered whether *B. subtilis* 3610 would also exhibit this behavior. Under the same media conditions (TSB with 1% glucose) *B. subtilis* 3610 exhibited visible pellicle formation and did not appear to form any submerged surface-associated biofilm (Fig. S5). The pellicles formed under these conditions were less robust and less wrinkled than in MSgg but were hydrophobic and could easily be manually removed from the liquid surface. The ability to form a pellicle in TSB appeared to be largely independent of glucose provision and was unaffected by treatment with proteinase K or DNaseI (Fig. S5). Furthermore, the pH of the growth medium was not found to decrease substantially during biofilm growth (data not shown).

## Discussion

The principal contribution of this investigation is the discovery that a plant-root-associated strain of *B. cereus* (905) can form two kinds of biofilms that are produced by seemingly unrelated mechanisms. One kind is pellicles, which form by a mechanism involving orthologs of genes employed by *B. subtilis* in pellicle formation, including *sinI* and the two *tasA*-like genes, *calY1* and *calY2*. Strikingly, however, *B. cereus* 905 can also form submerged, surface-associated biofilms by a process that has several of the hallmarks of *S. aureus* biofilm formation.

Although it has previously been observed that *B. cereus* strains can form biofilms, particularly in rings around the surface-air-liquid interface, the exact mechanism of biofilm formation had not been elucidated. We found that *B. cereus* 905 makes visible pellicles that float at the air–liquid boundary in a similar manner to that observed for *B. subtilis* 3610. Furthermore, by interrogating the *B. cereus* 905 genome we identified homologs for many of the genes that are important for this behavior in *B. subtilis*. The regulatory pathway leading to matrix production appears to be conserved between *B. subtilis* and *B. cereus* since both the master regulator Spo0A and the biofilm-specific regulator SinI were required for pellicle formation in *B. cereus* 905. However, the exact components of the biofilm matrix appear to differ. Two homologs for the biofilm matrix protein TasA appear to be required for pellicle formation in *B. cereus* 905 and might act redundantly. In earlier work, proteolytic activity was attributed to the protein encoded by *calY1* in *B. cereus* (camelysin) (Fricke et al. 2001; Grass et al. 2004). However, the shared sequence identity of these proteins with TasA, the synteny of the *calY* genes with *sipW* and *sinI/sinR,* and their importance for pellicle biofilm formation in *B. cereus* 905, suggests that they can act as matrix proteins that might be capable of exhibiting amyloid-like fiber formation (Romero et al. 2010). It was also shown that expression of both *tasA* homologs (*calY1* and *calY2*) in *B. cereus* group members *B. anthracis* and *B. thuringiensis* is under the control of the biofilm repressor SinR, reinforcing the idea that they play a role in biofilm formation (Pflughoeft et al. 2011; Fagerlund et al. 2014). Finally, in purifying CalY1 from *B. cereus* the authors noted that CalY1 was prone to SDS-resistant aggregation and was tightly attached to the cell wall (Fricke et al. 2001; Grass et al. 2004). This is reminiscent of the amyloid behavior displayed by TasA from *B. subtilis* (Romero et al. 2010). Further investigation of the function of the CalY proteins in the matrix will focus on whether these two proteins can functionally complement the lack of *tasA* in *B. subtilis*.

By contrast, the hypothetical EPS product of the 21-gene cluster identified in *B. cereus* did not appear to be important for pellicle formation, despite the essential nature of the equivalent product for *B. subtilis* biofilm formation. The EPS product made by *B. cereus* 905 might have another function, perhaps forming the capsule. If a second *eps* gene cluster exists in *B. cereus* we were unable to detect it by homology to the *B. subtilis* gene cluster. In agreement with our findings, it has been shown that unlike the *tasA-sipW* and *calY* genes of *B. anthracis* and *B. thuringiensis* which are under the control of SinI/SinR, as they are in *B. subtilis*, the equivalent *eps* gene clusters are not (Pflughoeft et al. 2011; Fagerlund et al. 2014). This might suggest that the *B. cereus* group of bacteria do not induce expression of *eps* under biofilm forming conditions and perhaps do not require EPS for biofilm formation.

As well as being able to make pellicles we also found that *B. cereus* can form a biofilm by a second, seemingly independent, mechanism. In complex medium supplemented with glucose *B. cereus* formed surface-associated submerged biofilms. These biofilms were not dependent on SinI or the TasA-like proteins, suggesting that another route to biofilm formation is important here. Although a small decrease in biofilm formation was seen in the Δ*calY1* mutant, the absence of a phenotype for the Δ*sipW* mutant suggests that CalY1 does not significantly contribute to matrix under these conditions, since SipW is required to process TasA to its mature form in *B. subtilis* matrix formation. This mode of biofilm formation was, however, dependent on the master regulator Spo0A, suggesting that it is closely tied to the physiological state of cells. Furthermore, we found that in forming these biofilms the pH of the growth medium decreased substantially. This pH decrease was dependent on provision of glucose and was required for biofilm formation. This is reminiscent of biofilm formation in the Gram-positive pathogen *S. aureus*. In *S. aureus* we suggested that the pH decrease that accompanies biofilm formation causes the accumulation of matrix protein and possibly DNA at the cell surface, which promotes the aggregation of cells (Foulston et al. 2014). We speculate that this might also be the case in *B. cereus* in which both a protein and DNA component of the matrix were found to be important for this form of biofilm. Efforts to test this idea directly have, however, been hampered by the relatively weak nature of these biofilms in *B. cereus*, making it difficult to determine the nature of the proteins in the matrix, as was possible for *S. aureus*; however, our genetic analysis indicates that neither of the TasA homologs CalY1 and CalY2 were essential for submerged biofilm formation.

Some undomesticated strains of *B. subtilis* can form thick submerged biofilms in complex media such as TSB (Bridier et al. 2011), suggesting that the ability to form submerged biofilms might be more widespread within the *Bacillus* group. One of these strains of *B. subtilis* (ND_medical_) was also found to form mixed biofilms with *S. aureus*, which could protect *S. aureus* against the action of biocides and this appeared to be mediated by the matrix material made by *B. subtilis* ND_medical_ (Bridier et al. 2012). This might suggest that under these conditions *B. subtilis* and *S. aureus* can share matrix material. The genetic requirements of biofilm formation in these undomesticated strains of *B. subtilis* were not investigated, and thus it remains unclear what matrix material is being utilized (Bridier et al. 2011, 2012). We were unable to observe such submerged, surface-associated biofilm in *B. subtilis* NCIB3610, which continued to form pellicle biofilms in complex media; however, this might reflect strain-specific differences or differences in the way biofilm assays were conducted. In addition to forming surface-associated biofilms the *B. subtilis* undomesticated strains also formed pellicles in complex media (Bridier et al. 2011). We did not find this to be the case with *B. cereus* 905, which exclusively made submerged biofilms in complex media.

Taken together we find that *B. cereus* can display at least two modes of biofilm formation dependent on environmental conditions. An interesting question that remains to be answered is whether both of these mechanisms of biofilm formation occur in nature and, if so, what role each of them plays in the biology of *B. cereus*. Of particular interest is which of these mechanisms is enacted in the context of the plant root and whether it is then important for conferring plant protection? Our genetic analysis of *B. cereus* biofilm formation sheds light on how a similar outcome, biofilm formation, can be achieved in different ways. On one hand, *B. cereus* 905 behaves in a similar manner to its relative *B. subtilis*, a harmless soil organism that also interacts with plants. On the other hand, *B. cereus* 905 can exhibit behaviors more in line with pathogenic bacteria, such as *S. aureus*. Most strikingly these two modes of behavior appear to be achieved in radically different ways and are likely operating under different environmental conditions.

## Supporting Information

**Figure S1.***Bacillus cereus* 905 Δ*spo0A* exhibits a sporulation defect. *B. cereus* 905 wild type and Δ*spo0A*::Km were grown in DSM medium at 37°C for 36 h. Cells were washed twice in PBS and spotted onto slides and covered with polylysine-treated cover slips. Slides were visualized by phase contrast microscopy with a 100× oil objective. **Figure S2.** Pellicle Formation can be complemented in *trans*. *B. cereus* 905 wild-type and deletion mutants with and without complementation constructs, as described in the text, were grown in MSgg for 3 days at 30°C in a 24 well plate and images were taken. **Figure S3.** Crystal violet stained biofilms of *Bacillus cereus* 905 in growth media of different pH. *B. cereus* 905 was grown in the indicated growth media in wells of a 12-well tissue culture plate at 37°C for 24 h. The medium was removed and biofilms were washed twice with PBS. The plates were stained with a 0.1% crystal violet solution for 30 min and then washed once with 1 mL of PBS. Plates were air dried before photographing. **Figure S4.** Submerged biofilm formation can be complemented in *trans*. *B. cereus* 905 and the indicated deletion mutants with and without complementation constructs, as described in the text, were grown in TSB with 1% glucose in wells of a 96-well tissue culture plate at 37°C for 24 h. The medium was removed and the OD_600_ measured (medium). The well surfaces were washed with PBS and the OD_600_ of the wash measured (wash). Finally cells on the surface of the well were removed by vigorous resuspension in PBS and the OD_600_ measured (biofilm). Values shown are the average of three independent experiments with error bars indicating the standard deviation. **Figure S5.** Pellicle Formation by *Bacillus subtilis* in a range of growth media. *B. subtilis* 3610 was grown in the indicated growth media for 24 h at 37°C in a 96-well plate before photographing pellicle formation. **Table S1.** Strains and plasmids used in this study. **Table S2.** Oligonucleotide primers used in this study. **Table S3.** Identification of *B. cereus* genes with similarity to genes known to be important for biofilm formation in *B. subtili*s. **Table S4.** A putative exopolysaccharide gene cluster in *B. cereus* 905.
