# Supplementary material for: Alternative modes of biofilm formation by plant-associated Bacillus cereus
Source: Microbiologyopen. 2015 Mar 31;4(3):452–64. doi: 10.1002/mbo3.251 (PMC4475387; doi:10.1002/mbo3.251)
Supplement: Supplementary file 1 — Figure S1.Bacillus cereus 905 Δspo0A exhibits a sporulation defect. B. cereus 905 wild type and Δspo0A::Km were grown in DSM medium at 37°C for 36 h. Cells were washed twice in PBS and spotted onto slides and covered with polylysine-treated cover slips. Slides were visualized by phase contrast microscopy with a 100× oil objective. Figure S2. Pellicle Formation can be complemented in trans. B. cereus 905 wild-type and deletion mutants with and without complementation constructs, as described in the text, were grown in MSgg for 3 days at 30°C in a 24 well plate and images were taken. Figure S3. Crystal violet stained biofilms of Bacillus cereus 905 in growth media of different pH. B. cereus 905 was grown in the indicated growth media in wells of a 12-well tissue culture plate at 37°C for 24 h. The medium was removed and biofilms were washed twice with PBS. The plates were stained with a 0.1% crystal violet solution for 30 min and then washed once with 1 mL of PBS. Plates were air dried before photographing. Figure S4. Submerged biofilm formation can be complemented in trans. B. cereus 905 and the indicated deletion mutants with and without complementation constructs, as described in the text, were grown in TSB with 1% glucose in wells of a 96-well tissue culture plate at 37°C for 24 h. The medium was removed and the OD600 measured (medium). The well surfaces were washed with PBS and the OD600 of the wash measured (wash). Finally cells on the surface of the well were removed by vigorous resuspension in PBS and the OD600 measured (biofilm). Values shown are the average of three independent experiments with error bars indicating the standard deviation. Figure S5. Pellicle Formation by Bacillus subtilis in a range of growth media. B. subtilis 3610 was grown in the indicated growth media for 24 h at 37°C in a 96-well plate before photographing pellicle formation. Table S1. Strains and plasmids used in this study. Table S2. Oligonucleotide primers used in this study. Table [file mbo30004-0452-sd1.pdf]

Supplemental Material

Supplemental Table 1. Strains and plasmids used in this study

Strains	Details	Reference/Source
B. cereus		
905	wild type, isolated from wheat rhizosphere	(Wang *et al.*, 2007)
TG001	*spo0A*::Km^r	This study
TG003	*sipW*::Km^r	This study
TG004	*sinI*::Km^r	This study
TG005	*calY1*::Km^r	This study
TG006	*calY2*::Km^r	This study
TG007	*calY1-Y2*::Km^r	This study
TG063	*eps,* unmarked mutant of 905	This study
TG008	*spo0A*::Km^r with complementation plasmid p78*spo0A*	This study
TG009	*calY1*::Km^r with complementation plasmid p78*calY1*	This study
TG088	*calY2*::Km^r with complementation plasmid p78*calY2*	This study
TG097	*sipW*::Km^r with complementation plasmid p78*sipW*	This study
TG098	*sinI*::Km^r with complementation plasmid p78*sinI*	This study
E. coli		
DH5α		Life Technologies
Bacillus subtilis		
NCIB 3610	Biofilm forming strain of *B. subtilis*	(Branda *et al.*, 2001)
**Plasmids**	**Details**	**Reference/Source**
pEBS	*Bacillus* suicide plasmid; Em^r	(Wang *et al.*, 2007)
pMarA	PCR cloning vector; Km^r, Ap^r, Em^r	(Breton *et al.*, 2006)
pEBSK	pEBS containing the *Km^r* gene from pMarA on a 1,048-bp BamHI-PstI fragment	This study
pMAD	Shuttle vector for allele replacement; Ap^r (*E. coli*) Em^r (*Bacillus*); containing *bgaB* gene encoding a thermostable β-galactosidase	(Arnaud *et al.*, 2004)
pGFP78	Shuttle vector for *Bacillus* and *E. coli*; Amp^r, Tet^r; containing F78 promoter screened from *B. subtilis* ISW 1214 genomic DNA and GFP gene	This study
pHY78	Shuttle vector for *Bacillus* and *E. coli*; Amp^r, Tet^r; containing F78 promoter screened from *B. subtilis* ISW 1214 genomic DNA	This study
pEBSKΔ*spo0A*	967-bp upstream and 1,094-bp downstream of *spo0A* gene PCR products amplified from *B.*	This study

	cereus 905 using the primers spo0A-Up-F/spo0A-Up-R and spo0A-Dn-F/spo0A-Dn-R, respectively, cloned into the pEBSK plasmid	
pMADΔ*spo0A*	3,072-bp Up-Km-Dn BglII-MluI fragment from pEBSKΔ*spo0A* cloned into BglII-MluI site of pMAD	This study
pEBSKΔ*sipW*	951-bp upstream and 1,027-bp downstream of *sipW* gene PCR products amplified from *B. cereus* 905 using the primers sipW-Up-F/sipW-Up-R and sipW-Dn-F/sipW-Dn-R, respectively, cloned into the pEBSK plasmid	This study
pMADΔ*sipW*	3,042-bp Up-Km-Dn BglII-MluI fragment from pEBSKΔ*sipW* cloned into BglII-MluI site of pMAD	This study
pEBSKΔ*sinI*	1,005-bp upstream and 712-bp downstream of *sinI* gene PCR products amplified from *B. cereus* 905 using the primers sinI-Up-F/sinI-Up-R and sinI-Dn-F/sinI-Dn-R, respectively, cloned into the pEBSK plasmid	This study
pMADΔ*sinI*	2,819-bp Up-Km-Dn BglII-MluI fragment from pEBSKΔ*sinI* cloned into BglII-MluI site of pMAD	This study
pEBSKΔ*calY1*	962-bp upstream and 1,107-bp downstream of *calY1* gene PCR products amplified from *B. cereus* 905 using the primers calY1-Up-F/calY1-Up-R and calY1-Dn-F/calY1-Dn-R, respectively, cloned into the pEBSK plasmid	This study
pMADΔ*calY1*	3,134-bp Up-Km-Dn BglII-MluI fragment from pEBSKΔ*calY1* cloned into BglII-MluI site of pMAD	This study
pEBSKΔ*calY2*	1,133-bp upstream and 955-bp downstream of *calY2* gene PCR products amplified from *B. cereus* 905 using the primers calY2-Up-F/calY2-Up-R and calY2-Dn-F/calY2-Dn-R, respectively, cloned into the pEBSK plasmid	This study
pMADΔ*calY2*	3,136-bp Up-Km-Dn BglII-MluI fragment from pEBSKΔ*calY2* cloned into BglII-MluI site of pMAD	This study
pEBSKΔ*calY1-Y2*	1,133-bp upstream and 1,107-bp downstream of *calY1-Y2* gene PCR products amplified from *B. cereus* 905 using the primers calY2-Up-F/calY2-Up-R and calY1-Dn-F/calY1-Dn-R, respectively, cloned into the pEBSK plasmid	This study
pMADΔ*calY1-Y2*	3,720-bp Up-Km-Dn BglII-MluI fragment from pEBSKΔ*calY1-Y2* cloned into BglII-MluI site of pMAD	This study
pMADΔ*eps*	909-bp upstream and 866-bp downstream of *eps* gene PCR products amplified from *B. cereus* 905 using the primers eps-P1/ eps-P2 and eps-P3/ eps-P4, respectively, cloned into the pMAD plasmid	This study

p78*spo0A*	831-bp *spo0A* fragment PCR products amplified from *B. cereus* 905 using the primers spo0A-F-C and spo0A-R-C cloned into the pGFP78 plasmid	This study
p78*calY1*	594-bp *calY1* fragment PCR products amplified from *B. cereus* 905 using the primers calY1-F-C and calY1-R-C cloned into the pGFP78 plasmid	This study
p78*calY2*	588-bp *calY2* fragment PCR products amplified from *B. cereus* 905 using the primers calY2-F-C and calY2-R-C cloned into the pGFP78plasmid	This study
p78*sipW*	570-bp *sipW* fragment PCR products amplified from *B. cereus* 905 using the primers sipW-F-C and sipW-R-C cloned into the pGFP78plasmid	This study
p78*sinI*	135-bp *sinI* fragment PCR products amplified from *B. cereus* 905 using the primers sinI-F-C and sinI-R-C cloned into the pHY78plasmid	This study

Supplemental Table 2. Oligonucleotide primers used in this study.

Target gene	Primer name	Primer sequence (5′-3′)
Km^R	Km_F	CGCGGATCCTCCGTCGATACTATGTTATACG
	Km_R	AACTGCAGTATGGACAGTTGCGGATGTAC
spo0A	spo0A-Up-F	GCTCTAGAACTCCACAAAAAGACAACGGTG
	spo0A-Up-R	CGCGGATCCTTCTCCGACATGTGTATCCTTC
	spo0A-Dn-F	AACTGCAGCCATGTCAAAAGCAAAACCTAC
	spo0A-Dn-R	CCGCTCGAGGATGCCTTTCCTACTAAAATCC
	spo0A-F	GAAGATCTACTCCACAAAAAGACAACGGTG
	spo0A-R	CGACGCGTGCCGTTCCTTCATCATTTAATA
	spo0A-F-C	GCTCTAGAAACAATTCATGTAGCCTAGAAG
	spo0A-R-C	CCCAAGCTTCAGCATACATCGGTTTTACTAA
sipW	sipW-Up-F	GCTCTAGAAATGGCTATGATTACGAAGGGTG
	sipW-Up-R	CGCGGATCCATCGCGTTGCTAATTATCTTCC
	sipW-Dn-F	AACTGCAGATTGACGGAGAAAAGAAAGATA
	sipW-Dn-R	CCGCTCGAGTTATGTTTCTCAGATTGCTCTT
	sipW-F	GAAGATCTATGGCTATGATTACGAAGGGTG
	sipW-R	CGACGCGTTTATGTTTCTCAGATTGCTCTT
	sipW-F-C	GCTCTAGAAAGGAGGAACTACTATGAAATTAATATGGAAGATAATTAGC
	sipW-R-C	CCCAAGCTTCTAGACGGATTGTTCTACTTTT
sinI	sinI-Up-F	GCTCTAGAAAGGAACATTTGCATTCTTTAGC
	sinI-Up-R	CGCGGATCCATAGGTTTGTTCTTTTTAACGA
	sinI-Dn-F	AACTGCAGTACAAATGTAATTCCTCCCTA
	sinI-Dn-R	AACTGCAGTTATCTAATTTTTCTTTCGTG
	sinI-F	CATGCCATGGAGGAACATTTGCATTCTTTAGC
	sinI-R	CGACGCGTCTAATTTTTCTTTCGTGTCTGC
	sinI-F-C	CCCAAGCTTAAGGAGGAACTACTTTGTACAAAGATAAGACAGACG
	sinI-R-C	CCCAAGCTTCTATTGAGCCTGACTGGAT
calY1	calY1-Up-F	GCTCTAGAAGTATGGCAACAAGGGCGTGAGA
	calY1-Up-R	CGCGGATCCCCCAATGCTGCTGATGCAACTC
	calY1-Dn-F	AACTGCAGCAACCAAGAAGCTGGGGAAGAA
	calY1-Dn-R	CCGCTCGAGCTGGGCTCGTAGCTGTTTTATT
	calY1-F	GAAGATCTGTATGGCAACAAGGGCGTGAGA
	calY1-R	CGACGCGTCTGGGCTCGTAGCTGTTTTATT
	calY1-F-C	GCTCTAGAGCTAGGGGGAATTGATTGTGAG
	calY1-R-C	CCCAAGCTTGCTTACAACCAGCACTTCTTTT
calY2	calY2-Up-F	GCTCTAGAAATCATAAAGGAATACATCGTGG
	calY2-Up-R	CGCGGATCCGCTCCTAATACTGCTGATGTAATA
	calY2-Dn-F	AACTGCAGTTGATGCACAACAAACAGCAGG
	calY2-Dn-R	CCGCTCGAGTTCTAGCTTTTTCTGTTCTTCC
	calY2-F	GAAGATCTATCATAAAGGAATACATCGTGG

	calY2-R	CGACGCGTTTCTAGCTTTTTCTGTTCTTCC
	calY2-F-C	GCTCTAGACGTTTGGTTATACTTTCCGTTT
	calY2-R-C	CCCAAGCTTTAGAAAAAGGGCTATCCATGTG
eps	eps-P1	CATTAACTAGACAGATCTATCGATGCATGCCATGGCAAGGGCTTCCATCACATCTAT
	eps-P2	GAAAAGAAAACCATTACGACAAGAATATTTCTTTCATCGCCACA
	eps-P3	TGTGGCGATGAAAGAAATATTCTTGTCGTAATGGTTTTCTTTTC
	eps-P4	CGATATCGGATCCATATGACGTCGACGCGTGCAATATGTCCAAGTGGTAAGA

Supplemental Table 3. Identification of *B. cereus* genes with similarity to genes known to be important for biofilm formation in *B. subtilis*.

***B. subtilis* 168 Gene**	***B. cereus* 905 Homolog**		**Closest Homolog of *B. subtilis* 168 Protein in *B. cereus* Autonomous Non-redundant Protein Sequence Database (taxid:86661)**		
**Gene Name (Locus Accession Number)**	***B. cereus* 905 Genome Annotation**	**% Amino Acid Identity Shared with *B. subtilis* 168 Homolog**	**Accession Number and Annotation**	**% Amino Acid Identity Shared with *B. subtilis* 168 Homolog**	**% Amino Acid Identity Shared with *B. cereus* 905 Homolog**
spo0A (BSU24220)	B.cereus_B905_ 4380	81	WP_000411415 chemotaxis protein CheY [Bacillus cereus]	82	97
tasA (BSU24620)	B.cereus_B905_ 1389 (*calY1*)	35	WP_016113888 SipW-cognate class signal peptide [Bacillus cereus]	35	80
tasA (BSU24620)	B.cereus_B905_ 1387 (*calY2*)	35	WP_016113888 SipW-cognate class signal peptide [Bacillus cereus]	35	59
sipW (BSU24630)	B.cereus_B905_ 1386	47	WP_000767814 signal peptidase [Bacillus cereus]	49	93
tapA (BSU24640)	N\A	N/A	N/A	N/A	N/A
sinI (BSU24600)	B.cereus_B905 _1392	38	WP_000276216 - SinI protein [Bacillus cereus]	41	93
sinR (BSU24610)	B.cereus_B905 _1391	68	WP_002188849 transcriptional regulator [Bacillus cereus]	68	95

The respective protein and corresponding locus tag from the *B. subtilis* 168 genome is indicated with the identified homologs from *B. cereus* 905 and the *B. cereus* group of bacteria (from the

autonomous non-redundant protein sequence database for this group (taxid:86661)). The percentage amino acid sequence identity shared between the indicated proteins is shown.

Supplemental Table 4. A putative exopolysaccharide gene cluster in *B. cereus* 905.

B. cereus 905 Gene	Predicted Function	Homolog in *B. cereus* ATCC 14579 Predicted *eps* Gene Cluster [1]	% Amino Acid Identity Between 905 and ATCC 14579
B.cereus_B905_ 5465	tyrosine-protein kinase	BC5279	99
B.cereus_B905_ 5464	capsular polysaccharide biosynthesis protein	BC5278	95
B.cereus_B905_ 5463	tyrosine-protein kinase	BC5277	97
B.cereus_B905_ 5462	tyrosine-protein phosphatase	BC5276	97
B.cereus_B905_ 5461	UTP-glucose-1-phosphate uridylyltransferase	BC5275	98
B.cereus_B905_ 5460	polysaccharide biosynthesis protein	BC5274	88
B.cereus_B905_ 5459	pyridoxal phosphate-dependent aminotransferase	BC5273	25
B.cereus_B905_ 5458	sugar transferase	BC5270	39
B.cereus_B905_ 5457	acetyltransferase	none	N/A
B.cereus_B905_ 5456	glycosyltransferase	none	N/A
B.cereus_B905_ 5455	poly(glycerol-phosphate) alpha-glucosyltransferase	none	N/A
B.cereus_B905_ 5454	Hypothetical protein	none	N/A
B.cereus_B905_ 5453	poly(glycerol-phosphate) alpha-glucosyltransferase	none	N/A
B.cereus_B905_ 5452	hypothetical protein	none	N/A
B.cereus_B905_ 5451	teichuronic acid biosynthesis protein	none	N/A
B.cereus_B905_ 5450	UDP-glucose 6-dehydrogenase	none	N/A
B.cereus_B905_ 5449	UDP-glucose epimerase	none	N/A
B.cereus_B905_ 5448	transcriptional regulator LytR	BC5265	87
B.cereus_B905_ 5447	Hypothetical protein	BC5264	92
B.cereus_B905_ 5446	UDP-glucose 4-epimerase	BC5263	40
B.cereus_B905_ 5445	UDP-glucose 4-epimerase	BC5263	99

The locus tag for each gene in the identified 21-gene cluster from *B. cereus* 905 is indicated. The predicted function of each encoded protein is given, based on the results of a BLAST analysis (NCBI) of similar proteins. The homologous protein, when applicable, encoded within the putative EPS biosynthetic gene cluster of *B. cereus* ATCC 14579 ([1] BC5279–BC5263 (Ivanova *et al.*, 2003)) is shown along with the percentage shared amino acid sequence identity.

Arnaud, M., Chastanet, A., De, M., and Débarbouillé, M. (2004) New vector for efficient allelic replacement in naturally nontransformable, low-GC-content, gram-positive bacteria. *Appl Environ Microbiol* **70**: 6887–91.

Branda, S.S., González-Pastor, J.E., Ben-Yehuda, S., Losick, R., and Kolter, R. (2001) Fruiting body formation by *Bacillus subtilis*. *Proc Natl Acad Sci U S A* **98**: 11621–6.

Breton, Y. Le, Mohapatra, N.P., Haldenwang, W.G., and Breton, Y. Le (2006) In vivo random mutagenesis of *Bacillus subtilis* by use of TnYLB-1, a mariner-based transposon. *Appl Environ Microbiol* **72**: 327–33.

Ivanova, N., Sorokin, A., Anderson, I., Galleron, N., Candelon, B., Kapatral, V., *et al.* (2003) Genome sequence of *Bacillus cereus* and comparative analysis with *Bacillus anthracis*. *Nature* **423**: 87–91.

Wang, Y., Wang, H., Yang, C.-H., Wang, Q., and Mei, R. (2007) Two distinct manganese-containing superoxide dismutase genes in *Bacillus cereus*: their physiological characterizations and roles in surviving in wheat rhizosphere. *FEMS Microbiol Lett* **272**: 206–13.

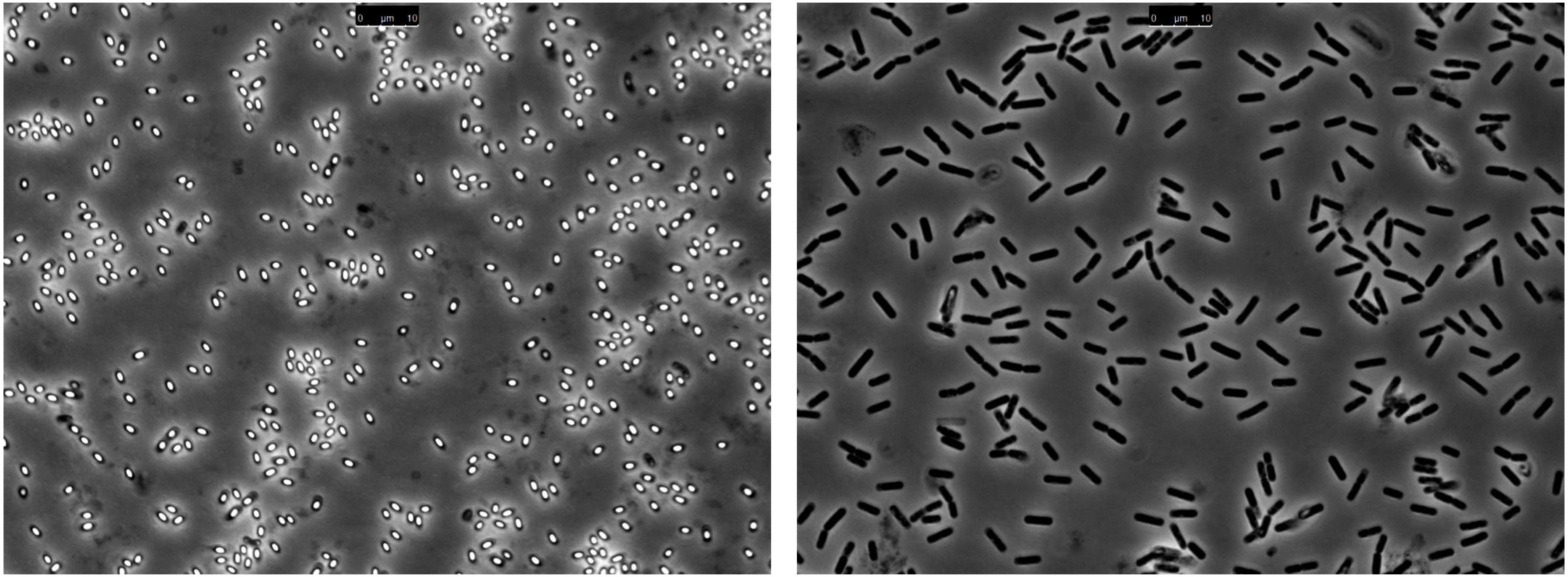

905 *Δspo0A*

Supplemental Figure 1. *B. cereus* 905 Δ*spo0A* exhibits a sporulation defect.
B. cereus 905 wild type and Δ*spo0A*::Km were grown in DSM medium at 37°C for 36h. Cells were washed twice in PBS and spotted onto slides and covered with poly-lysine treated cover slips. Slides were visualized by phase contrast microscopy with a 100x oil-objective.

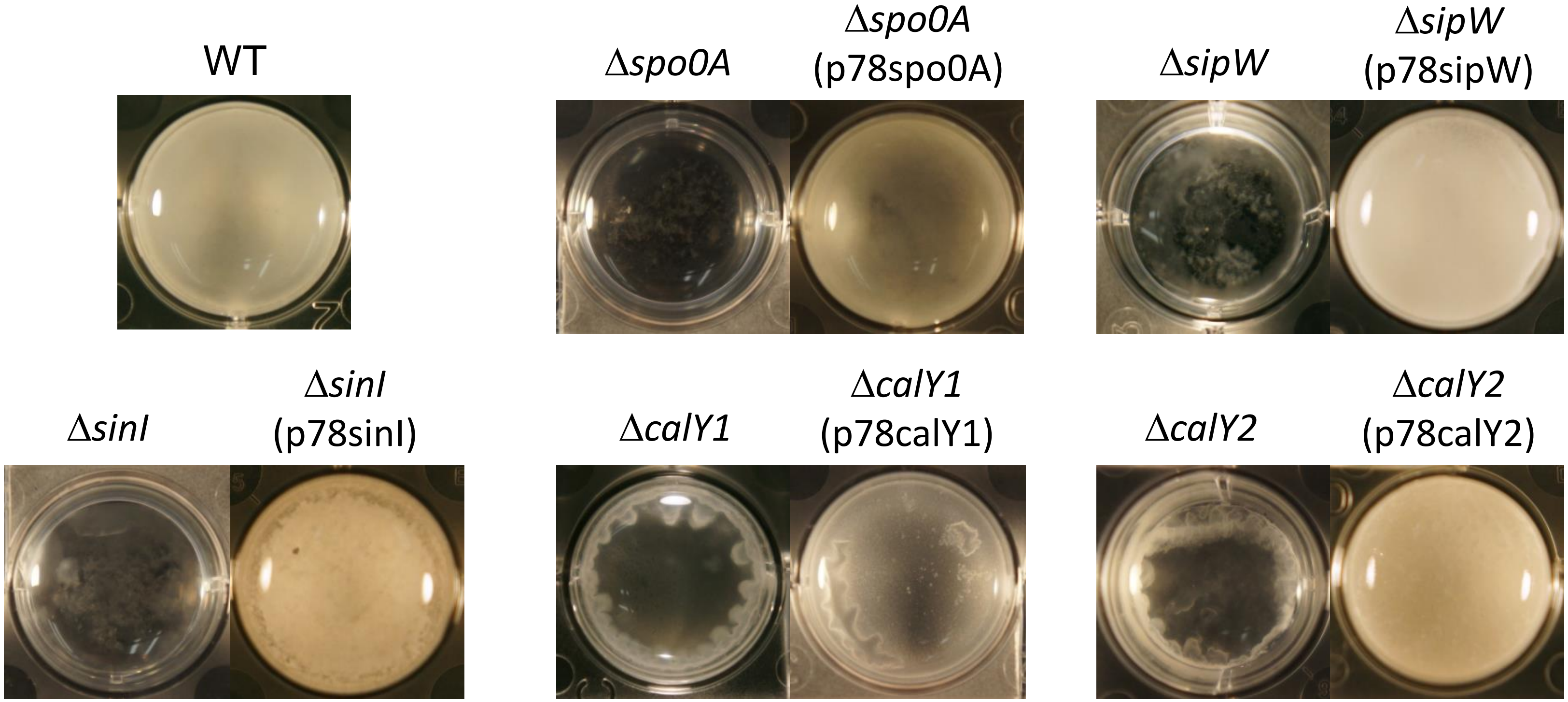

Supplemental Figure 2. Pellicle Formation can be complemented in *trans*.
B. cereus 905 wild type and deletion mutants with and without complementation constructs, as described in the text, were grown in MSgg for 3 days at 30°C in a 24 well plate and images were taken.

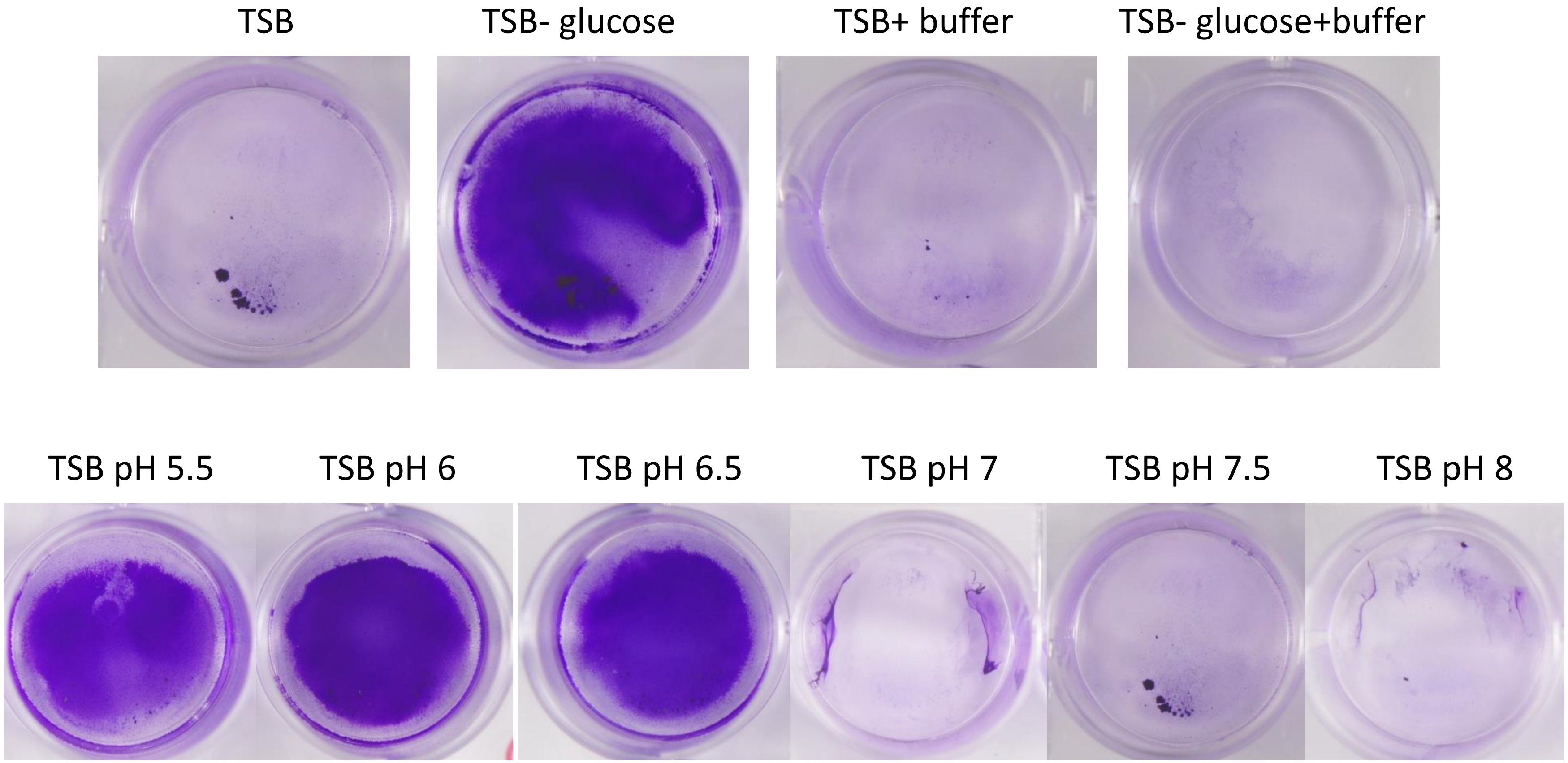

Supplemental Figure 3. Crystal violet stained biofilms of *B. cereus* 905 in growth media of different pH.
B. cereus 905 was grown in the indicated growth media in wells of a 12-well tissue culture plate at 37°C for 24h. The medium was removed and biofilms were washed twice with PBS. The plates were stained with a 0.1% crystal violet solution for 30 min and then washed once with 1 ml of PBS. Plates were air dried before photographing.

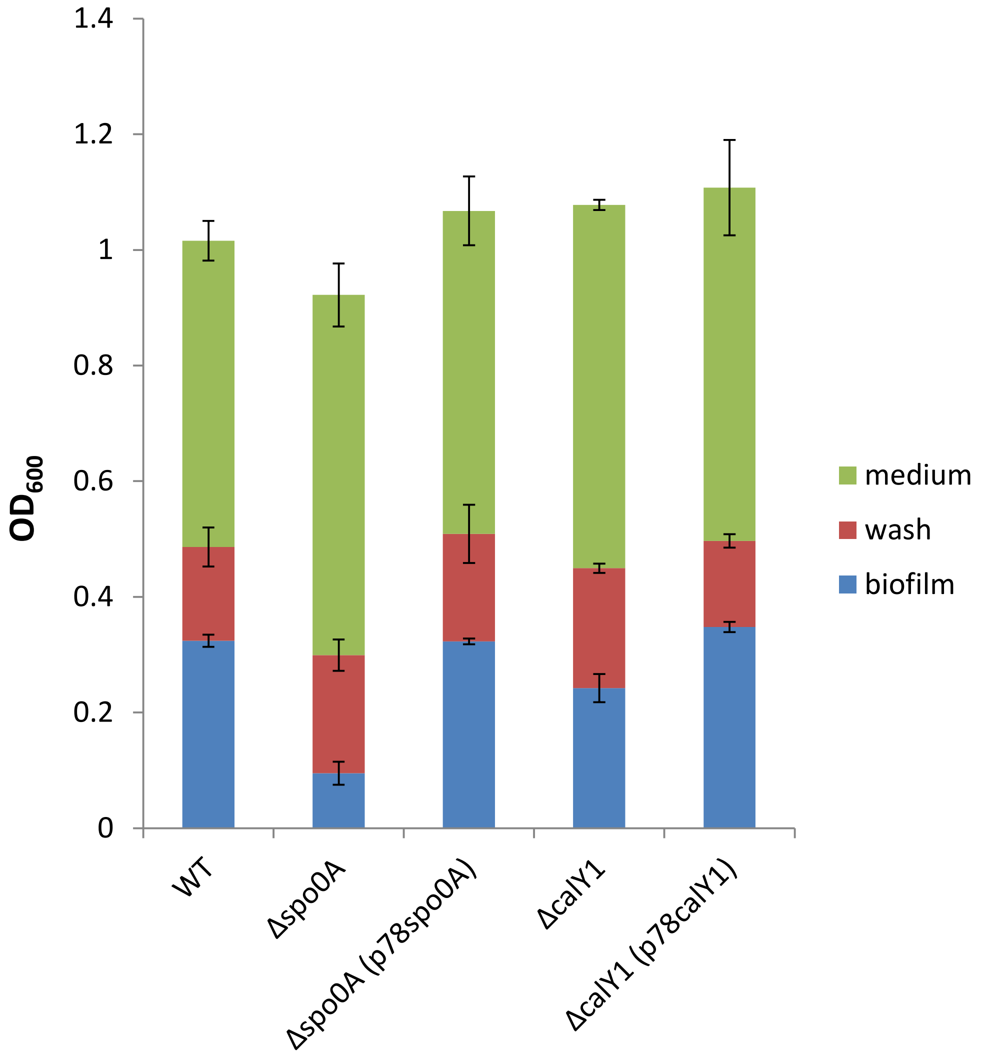

Supplemental Figure 4. Submerged biofilm formation can be complemented in *trans*.

B. cereus 905 and the indicated deletion mutants with and without complementation constructs, as described in the text, were grown in TSB with 1% glucose in wells of a 96-well tissue culture plate at 37°C for 24h. The medium was removed and the OD_{600} measured (medium). The well surfaces were washed with PBS and the OD_{600} of the wash measured (wash). Finally cells on the surface of the well were removed by vigorous resuspension in PBS and the OD_{600} measured (biofilm). Values shown are the average of three independent experiments with error bars indicating the standard deviation.

Bacillus subtilis 3610

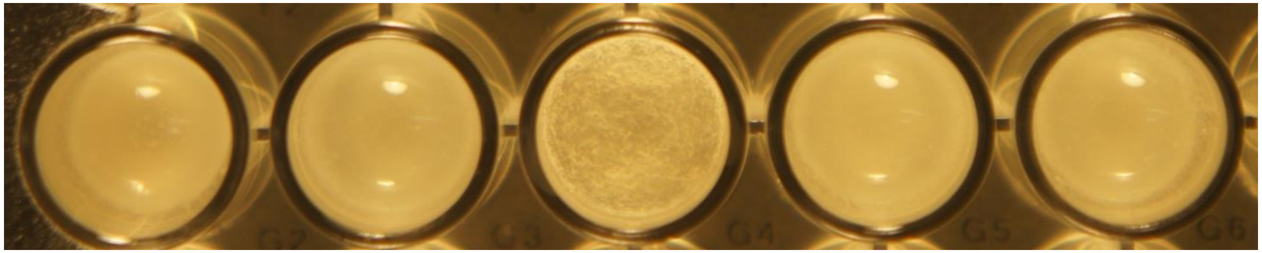

TSB | TSB+1%Glc | MSgg | TSB+1%Glc +PK | TSB+1%Glc +DNaseI

Supplemental Figure 5. Pellicle Formation by *B. subtilis* in a range of growth media.
B. subtilis 3610 was grown in the indicated growth media for 24h at 37°C in a 96-well plate before photographing pellicle formation.
